# Cryptococcal Meningitis in Young, Immunocompetent Patients: A Single-Center Retrospective Case Series and Review of the Literature

**DOI:** 10.1093/ofid/ofad420

**Published:** 2023-08-11

**Authors:** Matthew Stack, Jon Hiles, Ethan Valinetz, Samir K Gupta, Saira Butt, Jack G Schneider

**Affiliations:** Division of Infectious Diseases, Indiana University School of Medicine, Indianapolis, Indiana, USA; Division of Infectious Diseases, Indiana University School of Medicine, Indianapolis, Indiana, USA; Indiana University Health, Indianapolis, Indiana, USA; Division of Infectious Diseases, Indiana University School of Medicine, Indianapolis, Indiana, USA; Division of Infectious Diseases, Indiana University School of Medicine, Indianapolis, Indiana, USA; Division of Infectious Diseases, Indiana University School of Medicine, Indianapolis, Indiana, USA; Division of Infectious Diseases, Indiana University School of Medicine, Indianapolis, Indiana, USA

**Keywords:** cryptococcal, cryptococcus, fungal, meningitis, PIIRS

## Abstract

**Background:**

Cryptococcal meningitis is an uncommon but serious infection with high mortality and morbidity. Classically described in immunocompromised patients, including those with solid organ transplants or HIV/AIDS, cryptococcosis has also been reported in young and otherwise healthy patients, albeit rarely.

**Methods:**

We retrospectively searched for all cases of cryptococcal meningitis in young (≤50 years) and previously healthy patients with no known immunocompromising conditions from January 2015 to January 2022 at Indiana University Health (IU Health). Additionally, a PubMed literature review was performed with the keywords “cryptococcal meningitis” and “immunocompetent” from January 1988 to January 2022. Clinical courses, including outcomes and treatment regimens, were evaluated.

**Results:**

We identified 4 local cases of cryptococcal meningitis in otherwise healthy patients age ≤50 years. Three cases were due to *Cryptococcus neoformans,* with 1 experiencing a postinfectious inflammatory response syndrome (PIIRS). The PubMed search identified 51 additional cases, with 32 (63%) being caused by *Cryptococcus neoformans* and 8 (17%) by *Cryptococcus gattii*. Of the 51 cases, only 2 resulted in death directly due to cryptococcosis. Fifteen (29%) had PIIRS, with steroid treatment documented in 11 of 15. Antifungal induction regimens and duration were varied but predominately consisted of amphotericin and flucytosine, with a mean induction duration of 5.0 weeks.

**Conclusions:**

Cryptococcal meningitis in young, previously healthy patients is likely under-recognized. PIIRS (akin to immune reconstitution inflammatory syndrome observed in HIV/AIDS) with prolonged recovery should be of concern. Determining risk factors for cryptococcosis in these patients remains elusive.

Cryptococcal meningitis (CM) in immunocompetent patients is relatively uncommon but may not be as rare as previously thought. Some reports show that up to 20% of cases occur in immunocompetent individuals [1, 2]. Likewise, 1 single-center study stratifying patients with CM into HIV-positive, organ transplant recipient, and non-HIV/non–organ transplant groups showed that 36% of the cases were from the non-HIV/non–organ transplant group [3]. Another retrospective study that evaluated all cases of cryptococcosis at 2 large hospitals in Hong Kong found that 43.5% of the cases involved seemingly immunocompetent patients, 67% of whom presented with CM, many of whom had auto-antibodies [4]. All these reports likely still underestimate the true incidence and prevalence as providers may not have cryptococcosis high on their initial differential diagnoses in immunocompetent patients.

As the incidence of CM in patients with advanced HIV/AIDS continues to decline in the United States due to highly efficacious antiretroviral therapy (ART), a higher proportion of cases of CM in patients without HIV/AIDS is being described [5, 6]. Beyond HIV/AIDS and transplant, other risk factors for CM include patients with cirrhosis, poorly controlled type II diabetes, lupus, and chronic heavy alcohol use [7–11]. Other risk factors include an association with anti-granulocyte-macrophage colony-stimulating factor auto-antibodies and idiopathic CD4 lymphocytopenia [12–14]. However, studies focusing on cryptococcal meningitis in a population of young, previously healthy patients with no or only a few minor medical comorbidities are lacking. The primary aims of our study were to identify cases of CM in young and previously healthy individuals at our institution and described in the literature and summarize their clinical findings, including potential risk factors and treatment.

## METHODS

We retrospectively searched for all cases of CM in young, previously healthy patients with no or few minor comorbidities from January 2015 to January 2022 at Indiana University Health (IU Health) in Indianapolis, Indiana. No or few minor comorbidities was defined as either no significant medical history or a history of only 1 or a few minor comorbidities (such as gastroesophageal reflux disease or hypertension) that do not impair the immune system. All instances of blood or CSF cultures positive for *Cryptococcus* species and positive cryptococcal antigen titers were reviewed. We excluded cases in patients older than 50 years and excluded patients with immunosuppressing comorbidities (eg, cirrhosis, organ transplant, poorly controlled diabetes, sarcoidosis, etc.). We then performed a literature review of all CM cases in this patient population through a PubMed search with the keywords “cryptococcal meningitis” and “immunocompetent” from January 1988 until January 2022. We again excluded cases in patients older than 50 and patients with any major, possibly immunosuppressing comorbidities, articles not in English, and cases with isolated cryptococcoma but no associated meningitis.

For both the retrospective IU Health review and the PubMed review, we collected the following data with each case: sex, age, associated comorbidities, presenting symptoms, *Cryptococcus* species, treatment regimen(s), presence or absence of a postinfectious inflammatory response syndrome (PIIRS), if a steroid course was given for PIIRS, and reported follow-up. PIIRS was defined as outlined in the study by Anjum et al. [15]. If available, we collected precise treatment details, but comprehensive documentation of consolidation and maintenance therapy was missing in many cases.

### Ethical Review

Ethical review and approval were given by the Indiana University Institutional Review Board (IRB); our study was determined to be exempt by the IRB.

## RESULTS

We identified 4 cases of CM in otherwise healthy patients <50 years of age at IU Health from January 2015 to January 2022 (Table 1). All 4 cases occurred in males, and the average age (range) was 35.5 (24–44) years. Three cases were due to *Cryptococcus neoformans,* while 1 case had no growth, identified only by positive CSF cryptococcal antigen. The mean length of induction therapy was ∼3 weeks; specifically, the mean duration (range) of amphotericin was 23 (17–38) days and of flucytosine was 19 (12–22) days. Consolidation and maintenance therapy durations varied due to patients being readmitted for unrelated reasons and/or lack of clear follow-up or patient adherence; however, in all cases, the tentative plans were for ∼8–10 weeks of fluconazole 800 mg/d consolidation therapy followed by at least 1 year of fluconazole 200 mg/d maintenance therapy. The mean duration of consolidation and maintenance therapy combined was 24 weeks in our IU Health cohort (Supplementary Table 1). One patient experienced PIIRS, which was treated with corticosteroids and ruxolitinib, which is a Janus kinase inhibitor. Three of the 4 patients were eventually lost to follow-up; 1 patient was lost to follow-up immediately after discharge. There were no deaths reported, but 3 of the 4 experienced significant long-term sequelae (eg, symptoms persisting at least 3 months postdischarge). The 1 patient immediately lost to follow-up was excluded as long-term sequelae could not be assessed. While none of the patients had any known risk factors for acquiring cryptococcosis (eg, autoimmune disorders, cirrhosis, heavy alcohol use, etc.), 3 patients had a medical history notable for illicit substance use. Two of the patients had a history of active intravenous drug use, and 1 patient had a history of cocaine use (unclear route of use). Lastly, all patients in the IU Health cohort had negative HIV antigen/antibody testing, and 3 of the 4 had negative HIV viral RNA testing as well (1 patient had only antigen/antibody testing performed). Further details on the hospital courses and subsequent follow-up of the 4 patients are described in Supplementary Table 2.

**Table 1.** Clinical Characteristics of the Four Cases of Cryptococcal Meningitis at IU Health

Patient	Gender	Age	Comorbidities	Presenting Symptoms	Organism	Initial CSF CrAg Titer	Postinfectious Inflammatory Response Syndrome	Reported Follow-up
1	Male	44	History of illicit substance use (cocaine), asthma	RUQ pain, nausea, clay-colored stools, dark urine, headaches, and fever	*Cryptococcus neoformans*	Too high to calculate	No	Immediately lost to follow-up
2	Male	41	GERD, history of Bell's palsy	Bilateral headaches, photophobia, phonophobia, nausea, vomiting, chills, loss of appetite, generalized weakness, weight loss, dizziness, AMS	*Cryptococcus neoformans*	1:320	No	Residual left-sided weakness, balance issues, and vision issues, then lost to follow-up
3	Male	33	History of hepatitis C (untreated), history of acute hepatitis A (self-resolved), active illicit substance use (IVDU), bipolar disorder	Headaches, AMS, generalized weakness, falls, nausea, vomiting, weight loss	No growth	1:320	No	Psychiatric issues 1 y after diagnosis, then lost to follow-up
4	Male	24	Active illicit substance use (IVDU)	Nausea, vomiting, headaches, vision impairment, neck pain/stiffness, tinnitus	*Cryptococcus neoformans*	1:2560	Yes (treated with steroids and ruxolitnib)	Residual right-sided hearing loss

Abbreviations: AMS, altered mental status; CrAg, cryptococcal antigen; CSF, cerebrospinal fluid; GERD, gastroesophageal reflux disease; IVDU, intravenous drug use; RUQ, right upper quadrant.

**Figure 1.**
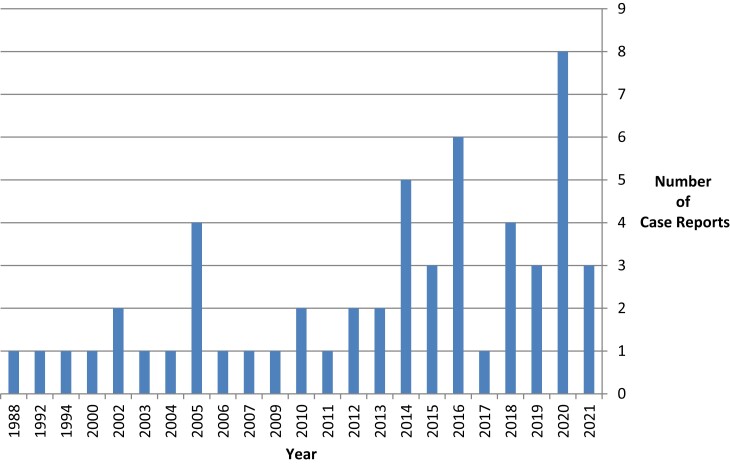
Number of case reports of cryptococcal meningitis in young, healthy individuals from January 1988 to January 2022.

The PubMed search identified 51 additional cases from January 1988 to January 2022 (Figure 1; Supplementary Table 3) [16–61]. Thirty-two cases were due to *Cryptococcus neoformans*, while 8 were due to *Cryptococcus gattii*. In 10 cases, the organism did not grow on culture or had no report. Species differentiation to *Cryptococcus neoformans* vs *Cryptococcus gattii* was missing in 1 case. Thirty-five cases occurred in males, and 16 cases occurred in females. The average age (range) was 35.5 (19–50) years. The presenting symptoms varied, consistent with cryptococcal meningitis, and there were no apparent immunosuppressant risk factors, although 10 cases did occur in patients with a history of illicit substance use.

Out of the 51 cases identified, only 2 deaths were attributed directly to CM. Fifteen of the 51 patients had PIIRS (29%), with steroid treatment documented in 11 of the 15 cases (73%). In addition to steroid treatment, there were a few reports of other adjunct anti-inflammatory therapy in which 1 patient received lenalidomide and another received interferon-gamma therapy.

There were 10 cases wherein patients did not have PIIRS but received antecedent steroid therapy for another indication before being diagnosed with CM. In almost all of those cases, steroids were used empirically for a suspected autoimmune disorder just before CM diagnosis. Apart from the 2 deaths reported, most patients were stated to be doing reasonably well at follow-up without significant long-term sequelae noted, but reported follow-up intervals were wide-ranging (range from 2 months to 3 years) and clear reporting of any lingering symptoms was lacking in some cases. Only 7.8% (4/51) were lost to follow-up. Lastly, antifungal treatment regimens and duration were varied, with the mean length for induction therapy being 5.0 weeks. The most common induction regimen consisted of amphotericin and flucytosine.

## DISCUSSION

The major findings of this study are that CM in young, previously healthy patients with no or only a few minor medical comorbidities is becoming increasingly recognized. We identified 4 such cases over a 7-year time period from January 2015 to January 2022 at our health system, IU Health, and identified 51 cases in the literature from January 1988 to January 2022. Most cases were due to *Cryptococcus neoformans* as opposed to *Cryptococcus gattii*. There were no clear risk factors except for a history of illicit drug use. Approximately one-third of the cases in the literature review reported a syndrome consistent with PIIRS, which required treatment with steroids. Most of the cases in the PubMed search were reported within the last 10 years, supporting the argument that this disease state is becoming increasingly recognized in young and otherwise healthy patients.

Typically, *Cryptococcus gattii* is thought to have a greater predilection for immunocompetent patients than *Cryptococcus neoformans* [62, 63]. However, our study found that the majority of patients both in the retrospective IU Health case series and the PubMed search had *Cryptococcus neoformans*. Similar to our findings, a prospective descriptive study of CM in HIV-uninfected patients in Vietnam without underlying comorbidities showed that most patients had *C. neoformans* [64]. These findings suggest that *Cryptococcus neoformans* may have a more significant role in immunocompetent patients than previously thought. In the PubMed literature search, only 2 patients died due to CM; 7.8% (4/51) were lost to follow-up. In the IU Health retrospective review, there were no deaths, although 3 of the 4 patients were lost to follow-up.

Determining risk factors for cryptococcosis acquired in otherwise healthy individuals still remains elusive. Evaluating why these young and otherwise healthy patients are affected by a disease primarily occurring in immunosuppressed individuals is imperative. In patients without HIV or an organ transplant, specific medical comorbidities such as cirrhosis, chronic heavy alcohol use, poorly controlled type II diabetes, and autoimmune disorders are known risk factors for CM. Other risk factors include an association with anti-granulocyte-macrophage colony-stimulating factor auto-antibodies and idiopathic CD4 lymphocytopenia. Our study, however, did not find any firmly conclusive risk factors in the young and otherwise healthy patient group, other than perhaps a history of illicit substance use (3 of the 4 patients identified in the IU Health cohort). Though most patients lacked any significant medical history in the PubMed literature search, the most identified comorbidity interestingly was illicit substance use, which occurred in 10 of the 51 patients (20%). Apart from a possible link with illicit/intravenous drug use, another possible risk factor could be rare, unidentified genetic immunodeficiencies, though this would require further investigation.

CM in young and otherwise healthy individuals with a history of intravenous drug use has been recently described in a few case reports in the literature [65–67] and also in a few older case reports from the 1990s [68, 69]. Additionally, there is evidence that illicit drug use can suppress/modulate the immune system [70–73]. Thus, it is not unreasonable to consider that patients actively using illicit drugs may be “relatively immunosuppressed.”

Whether or not illicit substance use is a real risk factor for young and otherwise healthy patients to acquire CM vs just a coincidental finding requires further investigation. Nonetheless, our findings combined with the multiple case reports in the literature do provide evidence of a possible link between intravenous drug use and young and otherwise healthy individuals acquiring CM. The retrospective nature of these case reports and our study preclude comprehensive epidemiological evaluations; a possible epidemiological link needs to be explored further via prospective cohort studies. Given that illicit drug use has become a serious and highly prevalent condition in the United States, this potential risk factor should further alert clinicians to consider *Cryptococcus* as a cause of meningitis in young and otherwise healthy persons.

Our study's main strength is that it is the first to attempt to comprehensively describe in detail CM in young and otherwise healthy patients with no or few medical comorbidities. In the literature, only 1 other review examines CM in immunocompetent patients [74]. However, that review provided only a more generalized overview of CM in immunocompetent individuals, whereas our study focused more on presenting symptoms, details on treatment regimens, presence/absence of PIIRS, and outcomes of each case in the literature of young and otherwise healthy patients with CM. An additional strength of this study is that the retrospective review of CM in young, healthy patients was performed at our home institution of IU Health, which is a large academic, tertiary care center (the only academic, tertiary care center in the state of Indiana) that sees a wide variety of pathology and a very diverse patient population. IU Health is comprised of 16 hospitals with about 2700 licensed beds. This allowed us to compare our cohort with those examined in the literature review.

The main weakness of this study is the descriptive, retrospective nature. Due to limited data provided from the included case reports, we cannot draw firm conclusions regarding risk factors, treatment regimens, or outcomes based on specific treatment regimens. Whether this study's observations would be corroborated in further investigations should be explored in larger, future studies. In addition, in the PubMed literature review, only 7.8% (4/51) of patients were reported as lost to follow-up. This number may be skewed lower given the limited data provided from the individual case reports and the variability in follow-up reporting. Finally, the retrospective review was limited in that it was only a single-center review and was restricted to 7 years.

Though this study offers a descriptive insight into CM in young and otherwise healthy patients, several unanswered questions remain. Arguably the biggest question is the best treatment regimen for this patient group. Unfortunately, there is a lack of data in the non-HIV, nontransplant group, and treatment recommendations are extrapolated from patients with HIV [75]. Recently, Ssebambulidde et al. did provide recommendations for non-HIV-associated cryptococcal meningoencephalitis, but the recommendations were still extrapolated mainly from patients with HIV [76]. Whether these patients should receive 2 vs 4 to 6 weeks of induction therapy is unclear. A recent study reported that a single high dose of amphotericin was noninferior to the current standard of care for patients with HIV with CM [77]. Data for induction therapy with a single high dose of amphotericin in this young and otherwise healthy patient group are unknown. Moreover, exactly how long this group of patients should be on maintenance therapy is also unclear. Unlike in patients with HIV, there is no clear surrogate for when the immune system recovers (eg, increasing CD4 count) in this patient group.

Lastly, a crucial, unresolved issue is the incidence and treatment of a postinfectious inflammatory syndrome akin to immune reconstitution inflammatory syndrome in patients with advanced HIV/AIDS, which has recently been named postinfectious inflammatory response syndrome [15]. A paradoxical postinfectious inflammatory response in previously healthy, non-HIV CM was first comprehensively described by Panackal et al., for which steroid treatment was given [77, 78]. In our literature review, 15 of 51 patients developed PIIRS, and 11 of the 15 cases (73%) received steroids. Anjum et al. also reported using steroids for PIIRS in previously healthy individuals with cryptococcal meningoencephalitis [15]. In that study, pulse corticosteroid taper therapy improved meningoencephalitis-related complications with minimal toxicity. In our brief literature review, there were 10 cases wherein patients did not have PIIRS but received antecedent steroid therapy for another indication (usually for a misdiagnosis of an autoimmune condition) just shortly before the formal diagnosis of CM. One could speculate that, in those cases, the patients did not experience PIIRS due to already being on or recently finishing a course of steroids.

## CONCLUSIONS

CM in young and otherwise healthy patients may not be as rare as previously thought, though it is still likely under-recognized. Thinking of this disease only in its textbook presentation in patients with advanced HIV/AIDS or other forms of immunosuppression may lead to missed diagnoses in otherwise healthy individuals. The possible link between intravenous drug use and cryptococcal meningitis is of paramount importance given the ongoing opioid epidemic in the United States. Because CM in young and healthy patients with no or few medical comorbidities is becoming increasingly recognized, further investigation is necessary to determine optimal treatment regimens and elucidate other potential risk factors in this specific patient population.

## Supplementary Material

ofad396_Supplementary_Data

## Supplementary Data

Supplementary materials are available at *Open Forum Infectious Diseases* online. Consisting of data provided by the authors to benefit the reader, the posted materials are not copyedited and are the sole responsibility of the authors, so questions or comments should be addressed to the corresponding author.
