# Supplementary material for: Cryptococcal Meningitis in Young, Immunocompetent Patients: A Single-Center Retrospective Case Series and Review of the Literature
Source: Open Forum Infect Dis. 2023 Aug 11;10(8):ofad420. doi: 10.1093/ofid/ofad420 (PMC10456216; doi:10.1093/ofid/ofad420)
Supplement: ofad396_Supplementary_Data [file ofad396_supplementary_data.zip › 2Supplem.docx]

| **Patient** | **Clinical Course** |
| --- | --- |
| Patient 1 | Patient was admitted twice during the initial course of disease; patient received steroid therapy during his first admission for suspected systemic mastocytosis vs hypereosinophlic syndrome vs autoimmune liver disease vs drug-induced liver injury (??) before cryptococcal meningitis was diagnosed very shortly after steroid therapy was started. Patient was continued on steroids during his second admission. Patient was readmitted for what ended up being unrelated issues, but initially thought to be azole toxicity so was briefly on amphotericin again but then changed back to fluconazole to finish 8 weeks. Patient immediately lost to follow up in 2015 after discharge from second admission. |
| Patient 2 | After patient's index admission, he was readmitted about 1.5 to 2 weeks later with fevers and AMS. Repeat LP was not really concerning for recurrence of cryptococcal meningitis; no concerns for a post-infectious inflammatory response syndrome (PIIRS) either. Patient was found to have positive blood cultures for Klebsiella pneumoniae on re-admission and this was felt to be the reason for patient’s symptoms (though source of the Klebsiella pneumoniae bacteremia was not clear). Patient did have a CD4 count drawn after a clinic follow up visit with Infectious Disease; interestingly, absolute CD4 count was 261 cells/μL and percentage was 25% but no follow up levels were obtained. Thus, it is possible that he could have had idiopathic CD4 lymphopenia but cannot confirm as patient had no follow up testing and had no other infectious syndromes to suggest an underlying immunodeficiency. Also of note, patient had a mild AKI on his index admission, so amphotericin was briefly held and replaced with high dose fluconazole. Lastly, the tentative plan was to continue maintenance therapy through week 63 but the patient stopped taking the maintenance Fluconazole at about week 22 (for unclear reasons) without recurrence. |
| Patient 3 | Patient presented to the ER shortly after discharge from his index admission and there was some question of adherence to PO fluconazole. The tentative plan was for high-dose consolidation therapy for 2 months then suppression for 1 to 2 years. Patient was not on fluconazole 1 year later on an admission for an unrelated issue. Patient appeared to be experiencing homelessness and struggled with substance abuse issues, so difficult to assess adherence. In addition, patient had significant psychiatric issues after discharge that further complicated assessing adherence and follow up. This patient was also ultimately lost to follow up unfortunately. |
| Patient 4 | Initially, the plan was for 4 weeks of induction therapy, but patient refused and left AMA about 3 weeks of induction therapy initially. After the patient's index admission, he was readmitted for about a month for what was ultimately determined to be PIIRS. Patient was started on steroids at that time. He was admitted again about a month later for bilateral lower extremity pain (etiology unclear; no changes to fluconazole or prednisone at that time). Unfortunately, patient was dealing with legal issues related to drug charges but reportedly was able to follow up with the NIH in 7/2022 for further management of PIIRS; appears the NIH started patient on ruxolitinib (which is a Janus-kina inhibitor). Patient is still dealing with legal issues that are complicating follow up. |
| Abbreviations: AMS=altered mental status; LP=lumbar puncture; AKI=acute kidney injury; ER=emergency room; PO=oral; AMA=against medical advice; NIH=National Institutes of Health. | |

**Supplementary Table 2:** Additional information about the hospital courses for the four patients in the Indiana University Health cohort.
