# Supplementary material for: Cryptococcal Meningitis in Young, Immunocompetent Patients: A Single-Center Retrospective Case Series and Review of the Literature
Source: Open Forum Infect Dis. 2023 Aug 11;10(8):ofad420. doi: 10.1093/ofid/ofad420 (PMC10456216; doi:10.1093/ofid/ofad420)
Supplement: ofad396_Supplementary_Data [file ofad396_supplementary_data.zip › 3Supplem.docx]

**Supplementary Table 3:** List and details of all case reports of cryptococcal meningitis in young and healthy individuals from January 1988 to January 2022, listed in order of publication date.

| **Citation** | **Year** | **Gender** | **Age** | **Comorbidities** | **Presenting Symptoms** | ***Cryptococcus* Species on CSF Culture** | **Treatment** | **Post-infectious Inflammatory Response Syndrome (PIIRS)** | **Reported Follow Up** |
| --- | --- | --- | --- | --- | --- | --- | --- | --- | --- |
| Correa et al. [33] | 2021 | Male | 40 | None | AMS, right-sided facial droop, unsteady gait, left temporal headache with associated dental pain | *C. neoformans* | —Induction: Amphotericin and 5FC for 8 wk  —Consolidation: PO Fluconazole for 12 wk —Maintenance: PO Fluconazole for 12 mo | No | Doing well other than continued headache symptoms |
| Reynard et al. [34] | 2021 | Male | 39 | None | Unsteady gait, trouble with speech, headaches, fatigue, fevers, vertigo, hypoacusis, personality changes | *C. neoformans* | —Induction: Amphotericin and 5FC for 3 wk  —Consolidation: IV Fluconazole (exact duration of IV Fluconazole not reported)  —Maintenance: PO Fluconazole for 10 mo | No | Completely asymptomatic at 2 years |
| Myers et al. [35] | 2021 | Female | 23 | Polysubstance use/IV drug use, chronic hepatitis C, depression, anxiety | AMS, posterior headache | *C. neoformans / gattii* | —Induction: Amphotericin and 5FC for 4 wk  —Consolidation: NR —Maintenance: PO Fluconazole (unclear duration lost to follow up) | No | Lost to follow up |
| Murphy et al. [36] | 2020 | Male | 35 | None | Throbbing headache | *C. neoformans* | —Induction: Amphotericin and 5FC for 4 wk  —Consolidation: Planned for PO Fluconazole for 8 wk but readmission 2 wk after discharge and restarted on induction therapy  —Maintenance: Planned for PO Fluconazole for 12 mo but readmitted as noted above | Yes (treated with steroids) | Was still hospitalized for PIIRS at time of publication |
| Raman et al. [37] | 2020 | Male | 38 | None | Fever, cough, lethargy, RUE and bilateral lower extremity weakness, aggressive behavior | NR | Antifungal treatment was reported as being given, but specific details not provided | No | Slowly recovering with residual weakness and intermittent headaches |
| Tanu et al. [38] | 2020 | Male | 34 | Well-controlled diabetes not requiring any medication | Fever, headaches, recurrent episodes of transient self-limited hemiparesis | *C. neoformans* | —Induction: Amphotericin and 5FC for 2 wk (but induction therapy was restarted for 6 wk when re-evaluated for PIIRS) —Consolidation: Initially PO Fluconazole for 2 wk (but after completing repeat induction therapy given for 8 wk)  —Maintenance: PO Fluconazole for 12 mo | Yes (treated with steroids) | Doing well at 2 years |
| Andreou et al. [39] | 2020 | Female | 31 | None | Fever, headaches, vomiting, AMS | *C. gattii* | —Induction: Initially Amphotericin and Fluconazole for 9 wk (but induction therapy of Amphotericin and Voriconazole was restarted when re-evaluated for PIIRS)^1^ —Consolidation: PO Fluconazole for 3 wk —Maintenance: PO Voriconazole (exact duration not reported) | Yes (treated with steroids) | Doing well at 18 months |
| Kathiresu et al. [40] | 2020 | Female | 41 | Remote history of hepatitis C | Occipital headaches, dizziness, visual disturbances, vomiting, unintentional weight loss | *C. neoformans* | —Induction: Amphotericin and 5FC for 6 wk  —Consolidation: NR —Maintenance: PO Fluconazole (exact duration not reported) | Yes (treated with steroids) | Symptoms significantly improved other than continued severe vision impairment |
| Polk et al. [20] (case 1) | 2020 | Male | 26 | IV drug use | Headache, blurred vision, AMS, seizures | *C. neoformans* | —Induction: Amphotericin and 5FC for 6 wk^2^ —Consolidation: PO Fluconazole (duration not reported) —Maintenance: PO Fluconazole (duration not reported) | Yes (treated with steroids) | Still with significant neurological deficits |
| Polk et al. [20] (case 2) | 2020 | Male | 30 | IV drug use, chronic hepatitis C | Headache, blurry vision, AMS, hearing loss, gait imbalance, dizziness | *C. neoformans* | —Induction: Amphotericin and 5FC (duration not reported) —Consolidation: NR —Maintenance: NR | No | Death from worsening cryptococcal meningitis |
| Jha et al. [41] | 2019 | Female | 50 | HTN, history of uterine fibroids (s/p TAH) | Vomiting, abdominal pain | Testing not performed | —Induction: Amphotericin and 5FC for 2 wk (induction therapy was delayed/interrupted due to socioeconomic issues in Nepal)  —Consolidation: PO Fluconazole for 8 wk —Maintenance: PO Fluconazole for 12 mo | No | Doing well at 2 months |
| Akyeampong et al. [42] | 2019 | Male | 30 | Marijuana use | New-onset seizures | No growth | NR | NR | NR |
| Hamdan et al. [43] | 2018 | Male | 40 | None | Headaches, vomiting | *C. neoformans* | —Induction: Amphotericin and 5FC for 4 wk  —Consolidation: PO Fluconazole for 8 wk —Maintenance: PO Fluconazole for 12 mo | No (but patient was given high-dose steroids shortly before the diagnosis of cryptococcal meningitis was made) | Doing well at 6 months |
| Zimelewicz-Oberman et al. [44] | 2018 | Female | 35 | Esophagitis, anorexia nervosa | Headaches, vomiting, blurred vision, vertigo, aphasia, gait instability, RUE paresthesia and weakness | *C. neoformans* | —Induction: Amphotericin and 5FC for 4 wk  —Consolidation: NR —Maintenance: PO Fluconazole for 10 wk | No (but patient was given steroids as part of induction treatment) | Continued vision impaired visual acuity |
| Carol et al. [45] | 2018 | Male | 19 | None | Acute low back pain and lower extremity weakness | *C. neoformans* | Antifungal treatment was reported as being given, but specific details not provided | N/A | Death from a nosocomial infection during initial hospitalization |
| Shapiro et al. [46] | 2018 | Female | 48 | HTN, marijuana use | Fatigue, dizziness, memory impairment, ataxia, left-sided numbness and weakness | *C. neoformans* | —Induction: Amphotericin and Fluconazole for 4 wk —Consolidation: NR —Maintenance: PO Fluconazole (exact duration not reported) | No | Doing well at time of hospital discharge |
| Ito et al. [47] | 2017 | Male | 49 | None | Anorexia, weight loss, increased skin pigmentation | No growth | —Induction: Amphotericin and 5FC for 2 wk  —Consolidation: PO Fluconazole (exact duration not reported)  —Maintenance: PO Fluconazole (still on maintenance therapy at time of publication) | No (but patient was already receiving steroids for adrenal insufficiency due to disseminated cryptococcosis) | Doing well several months after discharge |
| Chen et al. [48] | 2016 | Female | 45 | None | Headaches and neck stiffness | *C. gattii* | —Induction: Initially Amphotericin and 5FC for 4 wk (but induction therapy was restarted when re-evaluated for PIIRS) —Consolidation: PO Fluconazole for 12 wk —Maintenance: PO Fluconazole (exact duration not reported) | Yes (steroid treatment not given) | Doing well at 3 years |
| Li et al. [49] | 2016 | Female | 42 | None | Fatigue, blurred vision | *C. neoformans* | —Induction: Amphotericin and Fluconazole for 6 wk  —Consolidation: NR —Maintenance: PO Fluconazole for 8 wk | No (but patient was given high-dose steroids shortly before the diagnosis of cryptococcal meningitis was made) | Doing well at 3 months |
| Senadim et al. [50] | 2016 | Male | 19 | None | Frontal headache, diplopia | *C. neoformans* | —Induction: Amphotericin and Fluconazole for 13 wk  —Consolidation: NR —Maintenance: PO Fluconazole for 12 mo | Yes (steroid treatment not reported) | Continued memory impairment issues at 1 year |
| Amburgy et al. [51] | 2016 | Male | 45 | History of cocaine use | Headaches, fevers, chills, back pain, vomiting | *C. gattii* | —Induction: Amphotericin and 5FC for 12 wk —Consolidation: NR —Maintenance: PO Voriconazole (exact duration not reported) | Yes (treated with steroids and Interferon-gamma therapy) | Symptoms significantly improved at time of discharge |
| Shorman et al. [19] (case 1)^3^ | 2016 | Male | 29 | IV drug use, chronic hepatitis C | AMS | No growth | —Induction: Amphotericin alone? for 2 wk  —Consolidation: PO Fluconazole for 8 wk —Maintenance: NR | No | Lost to follow up |
| Shorman et al. [19] (case 2) | 2016 | Male | 39 | IV drug use, chronic hepatitis C, history of cerebral aneurysm (s/p clipping), history of latent TB | Headache, pain in lower extremities, inability to move lower extremities | *C. neoformans* | —Induction: Amphotericin and 5FC for 4 wk —Consolidation: NR —Maintenance: NR | No (but patient was given steroids for a presumed diagnosis of transverse myelitis shortly before the diagnosis of cryptococcal meningitis was made) | Lost to follow up |
| Arif et al. [52] | 2015 | Female | 21 | None | Headaches, weight loss, low-grade fevers | NR | —Induction: Amphotericin and 5FC for 6 wk  —Consolidation: NR —Maintenance: PO Fluconazole for 6 mo | No (but patient was given high-dose steroids shortly before the diagnosis of cryptococcal meningitis was made) | Doing well at several months after discharge |
| Merkler et al. [53] | 2015 | Female | 38 | None | Decreased visual acuity, dyschromatopsia | *C. neoformans* | —Induction: Amphotericin and 5FC for 4 wk —Consolidation: NR —Maintenance: PO Fluconazole for 12 mo | No (but patient was given high-dose steroids shortly before the diagnosis of cryptococcal meningitis was made) | Doing well at 1 year |
| Somerville et al. [54] | 2015 | Female | 36 | None | Headaches, ataxia, blurred vision, neck stiffness, nausea, vomiting | *C. neoformans* | —Induction: Amphotericin and 5FC for 8 wk  —Consolidation: NR —Maintenance: PO Fluconazole (still receiving at time of manuscript) | Yes (treated with both steroids and Thalidomide) | Symptoms improved at 18 mo follow up but persistent right leg spasticity was reported |
| Newsome & Nguyen [55] | 2014 | Male | 23 | Tinea versicolor, chronic sinusitis, benign ethmoid sinus osteoma | Vomiting, headache, weight loss, multiple syncopal episodes, nocturnal fevers | *C. neoformans* | —Induction: Amphotericin and Fluconazole and then 5FC and Fluconazole (exact total duration not reported) —Consolidation: PO 5FC and Fluconazole for 10 wk —Maintenance: None?/NR | No | Doing well at 10 weeks |
| Panigrahi et al. [56] | 2014 | Male | 36 | Tobacco use | Chest pain, cough with scant sputum, loss of appetite, unintentional weight loss | No growth | —Induction: Amphotericin and 5FC for 4 wk  —Consolidation: PO Fluconazole for 8 wk —Maintenance: PO Fluconazole for 14 mo | No | Doing well at 18 months |
| Niknam et al. [57] | 2014 | Female | 39 | None | Headaches, auditory and visual disturbances | *C. neoformans* | —Induction: Amphotericin and 5FC for 5 wk^4^ —Consolidation: Planned for a 8 week course but had to be discontinued due to drug-induced hepatitis  —Maintenance: NR | Yes (but patient was given steroids as part of induction treatment and also received steroids again when evaluated for recurrence of symptoms) | Doing well after being treated for recurrence of symptoms |
| Kawamura et al. [58] | 2014 | Male | 41 | None | Fever, cough, headache | *C. gattii* | —Induction: Amphotericin and 5FC for 8 wk  —Consolidation: PO Fluconazole for 8 wk —Maintenance: PO Fluconazole for 16 mo | No | Doing well at 1 year |
| Patil et al. [59] | 2013 | Male | 30 | None | Headaches, nausea, vomiting | *C. gattii* | —Induction: Amphotericin and 5FC for 2 wk  —Consolidation: PO Fluconazole for 8 wk —Maintenance: NR | No | Complete resolution of symptoms on follow up |
| Tabassum et al. [60] | 2013 | Female | 20 | History of tuberculous | Fever, cough, headache, vomiting, weight loss, umbilicated papules on head and neck region | *C. neoformans* | —Induction: Amphotericin for 2 wk  —Consolidation: PO Fluconazole for ~10 wk (but stopped taking Fluconazole after about 10 wk) —Maintenance: PO Fluconazole (still receiving at time of publication) | No (recurrence of symptoms was due to non-adherence to Fluconazole consolidation therapy) | Doing well at 8 mo follow up |
| Kaya et al. [61] | 2012 | Male | 46 | None | Headache, nausea, vomiting | *C. neoformans* | —Induction: Initially Amphotericin and 5FC for 2 wk (but induction therapy was restarted when re-evaluated for PIIRS) —Consolidation: NR —Maintenance: IV Fluconazole for 6 wk | Yes (treated with steroids and also had already received steroid treatment during induction therapy) | Doing well at follow up |
| Garber & Penar [62] | 2012 | Female | 27 | None | Occipital headaches, photopsia, nausea, vomiting | *C. neoformans* | —Induction: Amphotericin and 5FC for 2 wk  —Consolidation: PO Fluconazole for 6 wk —Maintenance: NR | No | Doing well at follow up |
| Gupta et al. [63] | 2011 | Male | 48 | None | Headaches, fevers, vomiting, AMS | *C. neoformans* | —Induction: Amphotericin until death 3 days later —Consolidation: N/A —Maintenance: N/A | N/A | Death from aspiration pneumonia |
| Singh et al. [64] | 2010 | Male | 32 | None | Fever, cough, dyspnea, loss of appetite, weight loss | NR | NR | NR | NR |
| Goldman et al. [65] | 2010 | Male | 25 | None | Seizures, headaches, nausea, photophobia, right hemianopsia, one episode of syncope | *C. gattii* | —Induction: Amphotericin and 5FC for 6 wk —Consolidation: PO Fluconazole (exact duration not reported) —Maintenance: NR | No | Doing well at 11 months with improved follow up imaging studies |
| Patro et al. [66] ^5^ | 2009 | Male | 23 | None | Headaches, double vision, slurred speech | No growth | Antifungal treatment was reported as being given, but specific details not provided | No | Doing well at one year |
| Mathews et al. [67] | 2007 | Male | 23 | None | Headaches, dizziness, nausea, vomiting, lethargy | *C. neoformans* | —Induction: Initially Amphotericin and 5FC for 2 wk (but induction therapy was restarted when re-evaluated for PIIRS) —Consolidation: PO Fluconazole for 5 wk —Maintenance: NR | Yes (treated with VP shunt; steroid treatment not mentioned) | Doing well at follow up |
| Agrawal et al. [68] | 2006 | Male | 38 | None | Low back pain, lower extremity paresis, bowel and bladder dysfunction | *C. neoformans* | Antifungal treatment with Amphotericin was reported as being given, but specific details were not reported | No (but patient was given steroids shortly before the diagnosis of cryptococcal meningitis was made) | Complete neurological recovery |
| Thompson, H.J. [69] | 2005 | Male | 46 | Tobacco use | Headache, nausea, fatigue, AMS | *C. neoformans* | —Induction: Amphotericin and 5FC for 6 wk (but induction therapy was restarted when re-evaluated for PIIRS) —Consolidation: PO 5FC for 10 wk —Maintenance: NR | Yes (treated with steroids) | Death18 mo after initial diagnosis due to a secondary infection |
| Lagrou et al. [70] | 2005 | Female | 44 | History of epilepsy | Headache, loss of appetite, nausea, weight loss | *C. neoformans* | —Induction: Amphotericin and 5FC for 2 wk  —Consolidation: NR —Maintenance: PO Fluconazole for 6 mo | No | Doing well after discharge |
| Lambertucci et al. [71] | 2005 | Male | 39 | None | Fever, headache, nausea, vomiting, photophobia | *C. neoformans* | —Induction: Initially Amphotericin and then switched to Fluconazole for 25 days (but induction therapy was restarted when readmitted for recurrence of symptoms)^6^ —Consolidation: NR —Maintenance: Amphotericin twice a week (exact duration not reported) | No (recurrence of symptoms was likely due to not being discharged on maintenance therapy) | Doing well after discharge while on outpatient Amphotericin |
| Lane et al. [72] | 2004 | Male | 29 | None | Headaches, neck stiffness, vomiting, and fevers | *C. gattii* | —Induction: Amphotericin and 5FC for 6 wk —Consolidation: PO Fluconazole (exact duration not reported) —Maintenance: PO Fluconazole for 9 mo | Yes (treated with steroids) | Doing well and completely asymptomatic at time of last follow up |
| Mansour et al. [73] | 2003 | Male | 23 | None | Anorexia, generalized weakness, occipital headache, neck stiffness, vomiting | *C. neoformans* | —Induction: Amphotericin for about 2 wk (was stopped after 2 wk due to side effects; subsequently switched to Fluconazole)  —Consolidation: PO Fluconazole for 11 wk —Maintenance: NR | Yes (but previously treated with steroids for Amphotericin injection reactions) | Doing well at 12 mo after Fluconazole was stopped |
| Taylor et al. [74] | 2002 | Male | 47 | None | AMS, cough, fever, weight loss | *C. gattii* | —Induction: Amphotericin and 5FC for 3 wk —Consolidation: NR —Maintenance: PO Fluconazole for at least 12 wk | No | Slowly improving at time of follow up after discharge |
| Kovoor et al. [75] | 2002 | Male | 44 | None | LLE weakness, memory loss, apathy, social disinhibition, urinary incontinences, vomiting, blurred vision, inability to speak | *C. neoformans* | Antifungal treatment was reported as being given, but specific details were not reported | No | Death at ~ 3 mo after diagnosis due to possible worsening hydrocephalus |
| Prendiville et al. [76] | 2000 | Female | 48 | Chronic sinusitis, history of bilateral maxillary antrostomies | Headaches, blurred vision, postnasal drip, generalized malaise, sixth cranial nerve palsy | *C. neoformans* | —Induction: Amphotericin and Fluconazole (exact duration not reported) —Consolidation: PO Fluconazole for 4 wk —Maintenance: Amphotericin for 8 wk | No (but given steroids shortly before the diagnosis of cryptococcal meningitis was made; recurrence of symptoms seemed to be due to inadequate treatment of concurrent skull base cryptococcal osteomyelitis ) | Doing well at follow up |
| Monno et al. [19] | 1994 | Male | 26 | IV drug use | Headaches, AMS, nuchal rigidity, photophobia | NR | —Induction: Amphotericin and 5FC for 4 wk —Consolidation: NR —Maintenance: PO Fluconazole for unclear duration | No (did have relapse of symptoms but this was due to nonadherence to maintenance therapy) | Reported some residual sequelae of infection but hard to determine as lost to follow up |
| Takeshita et al. [77] | 1992 | Male | 50 | None | Anorexia, weight loss, nausea, increased skin pigmentation | *C. neoformans* | —Induction: Combination of Amphotericin, 5FC, and Fluconazole for about 5 mo —Consolidation: NR —Maintenance: NR | No | Doing well after bilateral adrenalectomy |
| Stone et al. [78] | 1988 | Male | 39 | None | Decreased visual acuity of the right eye, headaches, photophobia, vomiting | *C. neoformans* | —Induction: Amphotericin and 5FC for 6 wk —Consolidation: NR —Maintenance: NR | No (but previously given steroids for presumed idiopathic posterior uveitis) | Doing well at 6 months after discharge |

Abbreviations: AMS=altered mental status; 5FC=flucytosine; NR=not reported; PO=oral; wk=weeks; mo=months; RUE=right upper extremity; HTN=hypertension; TAH=total abdominal hysterectomy; LLE=left lower extremity.

^1^Patient was briefly on 5FC initially, but was switched to fluconazole after 2 days due to suspected cardiotoxicity from flucytosine.

^2^Treated with a repeat 6 week course of amphotericin and 5FC on a subsequent admission though symptoms leading to readmission were likely driven mostly by PIIRS.^3^Shorman et al. described four cases in total. Two cases occurred in patients with a history of IV drug use; in the other two cases, one case was excluded due to patient being greater than 50 years of age and another was excluded due to a history of chronic heavy alcohol use.^4^During initial induction therapy, the patient was given a course of steroids due to persistence/worsening of symptoms. After initial induction therapy, patient was discharged on a course of PO fluconazole, but the fluconazole was stopped due to suspected drug-induced hepatitis. Patient was restarted on amphotericin and 5FC again for 8 wk but did not receive any maintenance therapy thereafter. After the 8 wk of repeat induction therapy, patient again developed neurological symptoms with CSF findings notable for an encapsulated yeast. The authors concluded this was a recurrence of infection given positive CSF findings, driven by patient not being on maintenance antifungal therapy.

^5^Patro et al. described two cases of cryptococcal meningitis in immunocompetent individuals, but we excluded one case as one of the individuals was greater than 50 years old.

^6^Patient was initially given conventional amphotericin for induction therapy but had to be switched to fluconazole monotherapy due to renal insufficiency, phlebitis, and disturbances in fluid/electrolyte balance.
