# Supplementary material for: Cryptococcal Meningitis in Young, Immunocompetent Patients: A Single-Center Retrospective Case Series and Review of the Literature
Source: Open Forum Infect Dis. 2023 Aug 11;10(8):ofad420. doi: 10.1093/ofid/ofad420 (PMC10456216; doi:10.1093/ofid/ofad420)
Supplement: ofad396_Supplementary_Data [file ofad396_supplementary_data.zip › 1Supplem.docx]

**Supplementary Table 1:** Antifungal treatment details of the four cases of cryptococcal meningitis at IU Health.

| **Patient** | **Amphotericin B** | | **5FC** | | **Fluconazole** | |
| --- | --- | --- | --- | --- | --- | --- |
| 1 | D1-12, D35-39 | 17 days | D1-12 | 12 days | D13-56 | 56 days |
| 2 | D1-5, D10-25 | 21 days | D2-26 | 25 days | D8-11, D26-289 | 268 days |
| 3 | D1-16 | 16 days | D1-16 | 16 days | D17-85 | 69 days |
| 4 | D1-21, D28-44 | 38 days | D1-19, D43-45 | 22 days | D22-306 | 285 days |
| **Mean Duration** | 23 days | | 19 days | | 169 days | |

Abbreviations: 5-FC=flucytosine; D=Hospital day (Day 1 is the first day of index hospitalization for CM)
